# Peak Systolic Velocity Ratio Cutoffs for Diagnosing Hemodynamically Significant Iliac Artery Stenosis: A Systematic Review and Evidence Gap Analysis

**DOI:** 10.7759/cureus.107166

**Published:** 2026-04-16

**Authors:** Elias Nabhan, Alexandrina-Paula Vana, Rida Khouri, Edouard Naoum Nehme, Nabil Poulos

**Affiliations:** 1 Cardiology, Centre Hospitalier Josephine Baker, Gonesse, FRA

**Keywords:** duplex ultrasound, hemodynamic significance, iliac artery stenosis, peak systolic velocity ratio, peripheral arterial disease

## Abstract

Peak systolic velocity ratio (PSVR) measured by duplex ultrasound has not been systematically compared against a physiologic reference standard. This systematic review and evidence gap analysis aimed to assess studies evaluating the validity of PSVR cut-offs in identifying hemodynamically significant iliac artery stenosis versus hyperemic intra-arterial (IA) pressure gradients. Five prospective studies with a total of 254 patients and 278 iliac artery segments from European vascular centers were identified in a comprehensive search of 7 databases from inception through August 6, 2025, with no language restriction. The heterogeneous hyperemic stimuli (papaverine, nitroglycerin, exercise) and heterogeneous reference thresholds were used in the studies and prevented pooling; hence, they were analyzed as four distinct target condition groups (Group A: ≥10 mmHg absolute; Group B: composite ≥20 mmHg/≥15% femoral-brachial index (FBI); Group C: ≥18% relative gradient; Group D: arteriographic). Using the most tested PSVR ≥ 2.5, sensitivity was 37%-83%, and specificity was 67%-93% between Groups A and B alone. Absolute PSV increase (DPSVe) ≥ 1.4 m/s after exercise was found to have 93% sensitivity and 87% specificity in the borderline-stenosis group. None of the studies had confidence intervals (CI). A structured sensitivity/specificity tabulation was used in the absence of reconstructable 2 x 2 data as a substitute for visual synthesis; formal meta-analysis was not possible. The quality of evidence was very low using the GRADE method. The main value of this review is to show that there is very limited and low-certainty evidence supporting physiologically validated PSVR thresholds; PSVR ≥ 2.5 should be considered an expert practice standard, rather than a validated diagnostic criterion.

## Introduction and background

Peripheral arterial disease (PAD) is one of the most common expressions of systemic atherosclerosis [1]. It affects 113 million individuals aged 40 and above in the world, and its prevalence increased by 72% between 1990 and 2019, according to the 2024 European Society of Cardiology (ESC) guidelines [1-2]. The iliac arteries are frequently involved, and early detection is essential to ensure appropriate treatment [3].

Duplex ultrasound is the main tool for non-invasive vascular assessment of PAD. The 2024 ESC guidelines recommend it as the first-line imaging modality to detect and localize PAD lesions [1]. It measures the peak systolic velocity ratio (PSVR) by dividing the peak systolic velocity (PSV) within a stenosis by the PSV in a nearby normal arterial segment just proximal to the stenosis [4-5]. This review specifically focuses on physiologic validation using pressure-based reference standards rather than anatomic imaging comparisons.

Although PSVR is commonly applied in clinical practice, there is still much controversy on the best cutoff value that can represent a hemodynamically significant lesion [1]. Cutoffs have been reported to be between 2.0 and 3.0, which shows that there is no standardization of the cutoff across vascular labs with clinical implications. If the hemodynamic significance of a stenosis is misclassified due to unreliable diagnostic thresholds, this may lead to both unnecessary invasive interventions and inappropriate deferral of clinically indicated revascularization [3-4].

One of the main problems in this field is the lack of consistency in how *hemodynamically significant* stenosis is defined. Existing studies use heterogeneous definitions of hemodynamic significance, complicating comparison across studies. In practical terms, what really matters is how much a lesion affects pressure and flow. The physiological significance of an iliac artery stenosis is determined by evaluating its effect on pressure and flow. The gradient of 10 mmHg or higher is frequently considered as a standard, but published values vary widely, between 5 and 20 mmHg [6-7].

This review intentionally focuses on physiological validity rather than anatomic correlation. Angiographic stenosis is not a reliable indicator of hemodynamic significance in aortoiliac disease, and research that compares PSVR to angiography measures a completely different endpoint. This is a methodological strength of the current review and the reason why there is a small number of studies included. To our knowledge, no systematic review has specifically evaluated PSVR thresholds for iliac artery stenosis validated against hyperemic intra-arterial (IA) pressure measurements. The purpose of this review is to point out the absence of physiologically validated thresholds and to describe the main methodological prerequisites for future research.

## Review

Methodology

The systematic review was conducted in accordance with the Preferred Reporting Items of a Systematic Review and a Meta-Analysis of Diagnostic Test Accuracy (PRISMA-DTA) guidelines [8]. The review was prospectively registered with PROSPERO (registration number CRD420261335044).

A thorough literature search was conducted on August 6, 2025, using 7 databases without language restriction, covering publications from database inception to the search date. Databases searched included MEDLINE (via PubMed), Embase (via Ovid), Cochrane Central Register of Controlled Trials (CENTRAL), Scopus, CINAHL via EBSCOhost, LILACS via the Virtual Health Library, and ClinicalTrials.gov. The search yielded 261 records before deduplication (PubMed 111, Embase via Ovid 51, Cochrane CENTRAL 14, Scopus 32, CINAHL 22, LILACS 2, ClinicalTrials.gov 29). Reference lists of included studies and relevant review articles were hand-searched for additional eligible publications. The reference standard concept block required terms denoting hyperemic IA pressure assessment (pressure gradient, hyperemia, papaverine, nitroglycerin). This review was designed to assess PSVR validation against a physiologic pressure reference standard. Including reference standard terms as a mandatory search concept was necessary to identify this distinct study type within a large duplex ultrasound literature. 

In this review, hemodynamically significant stenosis was considered as a hyperemic IA pressure gradient of 10 mmHg or more, because it was the most frequently used in eligible studies. The best physiologic threshold is still a controversial issue, and reported ranges are 5 to 20 mmHg [6-7]. Given the paucity of studies that applied a standardized physiologic threshold, we applied an inclusive eligibility criterion that retained studies that applied other but conceptually equivalent definitions of hemodynamic significance (relative pressure gradient, composite IA threshold, arteriographic classification with simultaneous pressure measurement). These are not pooled studies but rather analyzed as separate target condition groups with non-comparable reference standards.

The four groups are: Group A (≥10 mmHg absolute hyperemic gradient), Group B (composite threshold: ≥20 mmHg at rest or ≥15% femoral-brachial index (FBI) change post-papaverine), Group C (≥18% relative pressure gradient post-papaverine), and Group D (arteriographic ≥50% stenosis combined with femoral artery pressure (FAP) categories). Cross-group accuracy comparisons are not made. The sensitivity and specificity values mentioned in relation to PSVR ≥ 2.5 in this review are limited to studies in Groups A and B, which have a common construct of near-absolute pressure measurement.

The eligibility framework is summarized in Table 1.

**Table 1:** Eligibility criteria for literature inclusion. PSVR, peak systolic velocity ratio; IA, intra-arterial

Inclusion criteria	Exclusion criteria
Adult patients aged 18 years or older with suspected or confirmed iliac artery stenosis	Use of angiography alone as a reference standard without pressure measurement
Duplex ultrasound with PSVR calculation and a reported specific cutoff value	Use of resting pressure gradients only without hyperemic stimulation
IA pressure measurement under hyperemic conditions with a defined threshold for hemodynamic significance	Studies focusing exclusively on in-stent restenosis
Reporting diagnostic accuracy data for a specific PSVR cutoff	Studies focusing exclusively on endofibrosis in athletes due to a distinct pathophysiology
Studies using ≥10 mmHg hyperemic IA pressure gradient as threshold	Duplicate publications
Studies using alternative but conceptually equivalent definitions of hemodynamic significance (relative pressure gradient, composite IA threshold, or arteriographic classification with simultaneous pressure measurement)	

Two reviewers independently evaluated the full texts of eligible studies. The disagreements were solved by discussion or consultation with a third reviewer where necessary. Data were extracted by two reviewers through a standardized data extraction form. Extracted information included study characteristics, patient characteristics, index test details, reference standard details, PSVR cutoffs evaluated, diagnostic accuracy metrics, and unit of analysis (per patient or per segment).

Study quality was assessed using the QUADAS-2 tool [9]. Each included study was assessed by two reviewers on four domains: patient selection, index test, reference standard, and flow and timing. Spectrum bias was considered a source of high risk of bias, since this restriction may inflate or distort sensitivity and specificity estimates [10]. Blinding, threshold pre-specification, and handling of indeterminate results were also evaluated. The statistical consequences of per-segment and per-patient analysis were explicitly addressed; per-segment reporting does not take into account within-patient clustering and thus underestimates the variance and gives too narrow confidence intervals (CI).

Hierarchical models of meta-analysis were considered. It was not possible to reconstruct 2 x 2 contingency tables because: the raw data were not available in all five studies; per-segment non-independent observations that cannot validly populate contingency tables; and the reference standards across the target condition groups were fundamentally incomparable. It was considered uninformative and potentially misleading to plot sensitivity/specificity pairs in receiver operating characteristic (ROC) space since the five data points are based on four different target conditions.

In the absence of reconstructable 2 × 2 data, a tabulated range of sensitivity and specificity was employed as a surrogate to visual synthesis. This tabulation categorizes estimates by target condition group to explicitly indicate which comparisons are valid and is provided with a narrative synthesis. This methodology adheres to PRISMA-DTA recommendations on reviews in which formal pooling cannot be done and is the highest degree of quantitative synthesis that is possible with this evidence base. No inferential statistical comparisons were performed. Accordingly, all reported sensitivity and specificity estimates are interpreted descriptively rather than inferentially, and no statistical comparisons between studies are performed.

The overall quality of evidence was determined using the GRADE method for diagnostic test accuracy studies [11,12]. The evidence was evaluated in five domains, including risk of bias, inconsistency, indirectness, imprecision, and publication bias. The sensitivity and specificity were rated separately as high, moderate, low, or very low.

Results

**Figure 1:**
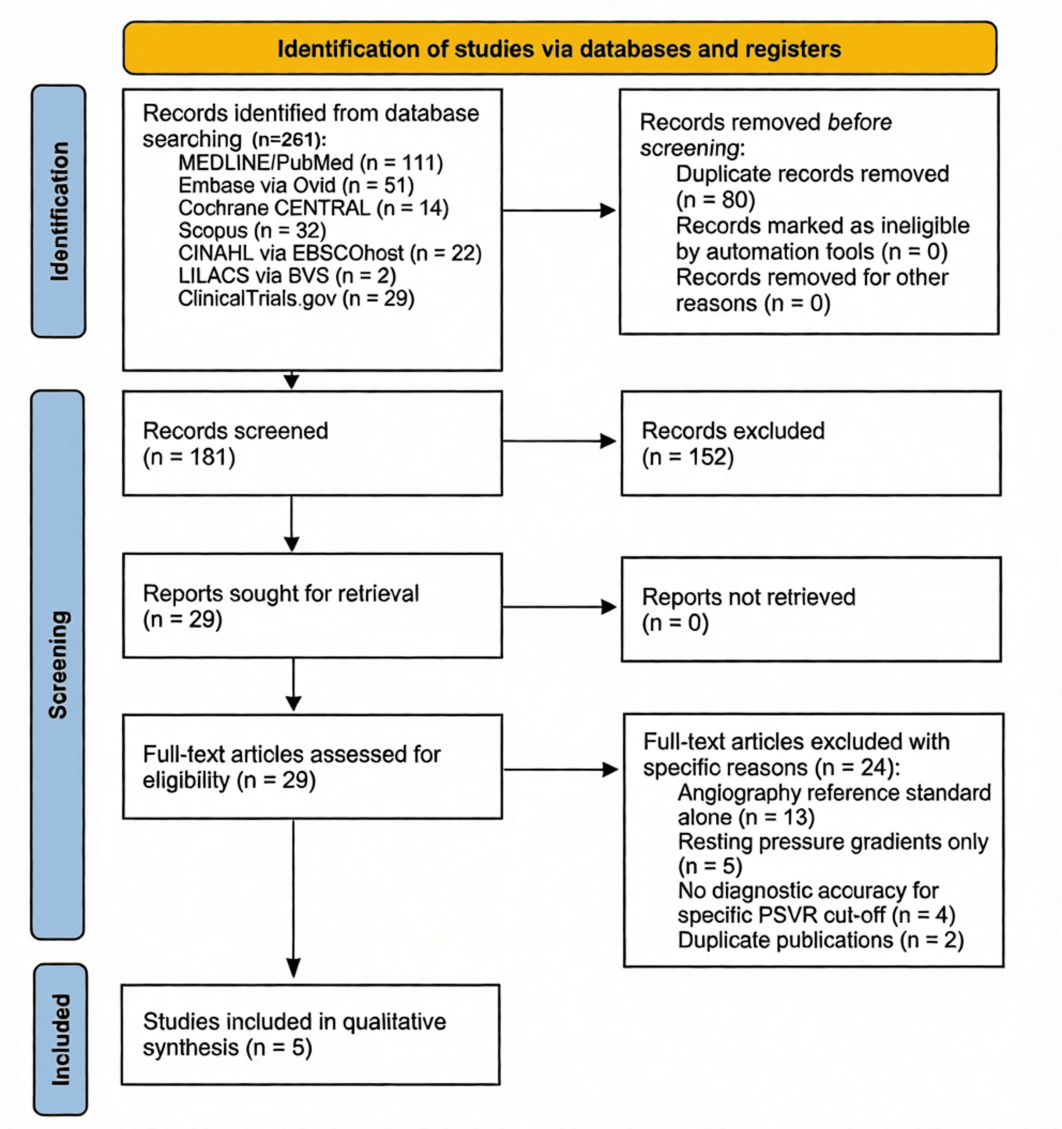
Study selection process using PRISMA 2020 open-access reporting guideline format. Source: [8]. PRISMA, Preferred Reporting Items for Systematic Reviews and Meta-Analyses

The initial database search yielded 261 records across 7 databases. After removal of duplicates, 181 unique titles and abstracts were screened; 152 were excluded at the title/abstract stage. Twenty-nine full-text articles were assessed for eligibility, of which 24 were excluded: 13 used angiography alone as a reference standard without pressure measurement, 5 used resting pressure gradients only without hyperemic stimulation, 4 did not report diagnostic accuracy for a specific PSVR cutoff, and 2 were duplicate publications. Five studies met all inclusion criteria: Heinen et al. [13], Coffi et al. [14], Coffi et al. [15], de Smet et al. [16], and Sensier et al. [17]. No additional studies were identified through hand-searching. The selection process was done using PRISMA 2020 open-access reporting guideline format (Figure 1) [8].

The five included studies were published between 1996 and 2018. Four originated from the Netherlands [13,14,15,16] and one from the United Kingdom (Leicester) [17]. All five were prospective cohort studies enrolling consecutive symptomatic patients with suspected iliac artery disease. A total of 254 patients and 278 iliac artery segments were evaluated. The unit of analysis was per segment in all studies.

Coffi et al. [15] and Coffi et al. [14] draw from substantially the same patient cohort at the Academic Medical Center, Amsterdam, both enrolling patients with PSVR 1.5-3.5. The 2002 paper is a more detailed analysis that includes exercise hyperemia and examines 43 of the 53 patients who survived in the 2001 study and underwent the exercise program. These two publications are considered as two separate eligible studies since they report different index tests, including resting PSVR and post-exercise absolute PSV increase (DPSVe), and they are in different target condition groups (A versus B); however, the overlap in their patients implies that they should not be counted twice in this review. Study characteristics are summarized in Table 2.

**Table 2:** Characteristics of included studies. PSVR, peak systolic velocity ratio; IA, intra-arterial; %, percentage; CLTI, chronic limb-threatening ischemia; FBI, femoral-brachial index; NR, not reported; FAP, femoral artery pressure; DPSVhyp, hyperemic PSV difference at stenosis; DFBI, absolute increase in femoral-brachial index

Study [Reference]	Year	Country	Patients (*n*)	Segments (*n*)	Hyperemic stimulus	Reference threshold	PSVR cutoffs	Patient spectrum/notes
Heinen et al. [13]	2018	The Netherlands	30	35	Nitroglycerin IA	≥10 mmHg (absolute)	2.5 (pre-specified)	Restricted: equivocal stenoses 30%-75% on angiography
Coffi et al. [14]	2002	The Netherlands	53 (43 analyzed)	58 (43 analyzed)	Bicycle ergometer exercise (2 W/kg × 2 min)	≥20 mmHg at rest OR ≥15% DFBI post-papaverine (composite)	DPSVe ≥1.4 m/s (primary); PSVR ≥2.0, ≥2.5 (comparison)	Restricted: PSVR 1.5-3.5 (borderline); overlaps with Coffi 2001 cohort
Coffi et al. [15]	2001	The Netherlands	53	58	Papaverine 50 mg IA	≥10 mmHg (absolute)	2.0, 2.5, 3.0	Restricted: PSVR 1.5-3.5 (subcritical); same cohort as Coffi 2002
de Smet et al. [16]	1996	The Netherlands	95	112	Papaverine (dose NR)	Arteriographic ≥50% stenosis + FAP pressure categories	2.8	Unselected consecutive symptomatic patients (claudication/CLTI)

Four out of five studies (except the study by de Smet et al. [16]) were assessed as high risk of patient selection because of spectrum restriction. Heinen et al. [13] considered patients with equivocal stenoses only (30%-75% on angiography). Both studies by Coffi et al. [14,15] preselected patients with PSVR 1.5-3.5, which is a small borderline range. Sensier et al. [17] chose patients whose velocity ratios were at least 1.5 and specifically targeted moderate disease at the PSVR 2.0 boundary, which added significant spectrum limitation. The exception was de Smet et al. [16], who recruited consecutive unselected symptomatic patients and received a low-risk rating on patient selection.

In the case of the index test domain, Heinen [13] and the two Coffi studies [14,15] were considered as low risk (pre-specified thresholds, blinding reported). de Smet et al. [16] was considered unclear (pre-specification of the threshold and blinding not clearly reported). Sensier et al. [17] was considered unclear since the PSVR ≥ 2.0 threshold was not prospectively registered but was taken over from the previous literature, and the ultrasonographer was not blinded to pressure outcomes. In the reference standard domain, Heinen and Coffi 2001 were in the low-risk category (≥10 mmHg absolute, pre-defined). The composite threshold resulted in Coffi 2002 being rated as unclear. de Smet et al. and Sensier et al. were rated unclear because of the arteriographic primary reference and the relative gradient threshold, respectively. The five studies all had low flow and timing risk. The results of QUADAS-2 are shown in Table 3.

**Table 3:** QUADAS-2 risk-of-bias assessment for included studies. L, low risk; U, unclear; H, high risk

Domain [Reference]	Heinen et al. [13]	Coffi et al. [14]	Coffi et al. [15]	de Smet et al. [16]	Sensier et al. [17]
Patient Selection	High	High	High	Low	High
Index Test	Low	Low	Low	Unclear	Unclear
Reference Standard	Low	Unclear	Low	Unclear	Unclear
Flow and Timing	Low	Low	Low	Low	Low

All five studies presented results per arterial segment rather than per patient, without taking into account within-patient clustering, in which multiple segments originate from the same individual. The main statistical implication is that it underestimates the variance and gives excessively narrow CI; point estimates can also be biased with respect to intra-patient correlation structure. The sensitivity and specificity estimates of all the included studies were not reported as CI, rendering any direct comparison of point estimates unreliable. The impact of this issue can be approximated. With an assumed moderate intra-patient correlation (intraclass correlation coefficient 0.3-0.5) and a mean of 1.1-1.3 segments per patient across these datasets, a design effect of about 1.1-1.2 reduces the effective sample size. Even in the smallest study included (Sensier et al., 23 patients, 25 segments), the corrected 95% CI around the presented sensitivity and specificity estimates would become significantly larger, and the point estimates would be statistically indistinguishable from a large set of other possible values. This is especially relevant to the interpretation of the 100% sensitivity of Sensier et al., which, even before clustering correction, has a binomial 95% CI of approximately 85%-100% with 20 true positives.

Diagnostic accuracy data for all five included studies, stratified by target condition group, are summarized in Table 4. These estimates should therefore be interpreted as descriptive ranges rather than precise measures of diagnostic performance.

**Table 4:** Diagnostic accuracy data stratified by target condition groups. Group A: ≥10 mmHg absolute; Group B: composite ≥20 mmHg/≥15% FBI; Group C: ≥18% relative; Group D: arteriographic. Sens, sensitivity; Spec, specificity; PPV, positive predictive value; NPV, negative predictive value; %, percentage; NR, not reported; IA, intra-arterial; papav., papaverine; nitro, nitroglycerin; DPSVe, absolute increase in PSV after exercise; DPSVhyp, hyperemic PSV difference at stenosis; ROC, receiver operating characteristic; DFBI, absolute increase in femoral–brachial index

Study [Ref]	Parameter and cutoff	Segments (*n*)	Sens (%)	Spec (%)	PPV/NPV (%)	Reference threshold	Notes/Target condition group
Heinen et al. (2018) [13]	PSVR ≥ 2.5	35	83	67	NR/NR	≥10 mmHg IA (nitro)	Group A (≥10 mmHg). Pre-specified; nitroglycerin; equivocal stenoses
Coffi et al. (2001) [15]	PSVR ≥ 2.5	58	37	90	NR/NR	≥10 mmHg IA (papav.)	Group A (≥10 mmHg). Borderline spectrum (PSVR 1.5-3.5)
Coffi et al. (2001) [15]	PSVR ≥ 2.0	58	75	80	NR/NR	≥10 mmHg IA (papav.)	Group A. From the ROC analysis in the original paper
Coffi et al. (2001) [15]	PSVR ≥ 3.0	58	39	93	NR/NR	≥10 mmHg IA (papav.)	Group A. From the ROC analysis in the original paper
Coffi et al. (2002) [14]	DPSVe ≥ 1.4 m/s	43	93	87	93/87	≥20 mmHg at rest OR ≥15% DFBI (papav.) (composite)	Group B (composite). Best-performing parameter; outperforms resting PSVR
Coffi et al. (2002) [14]	PSVR ≥ 2.0	43	75	80	88/63	≥20 mmHg at rest OR ≥15% DFBI (papav.) (composite)	Group B. Comparison arm within Coffi 2002
Coffi et al. (2002) [14]	PSVR ≥ 2.5	43	39	93	92/45	≥20 mmHg at rest OR ≥15% DFBI (papav.) (composite)	Group B. Comparison arm within Coffi 2002
Sensier et al. (2000) [17]	PSVR ≥ 2.0	25	100	21	NR/NR	≥18% relative gradient (papav.)	Group C (relative %). 100% sens but 21% spec; see caution note. Small n=23 pts.
Sensier et al. (2000) [17]	DPSVhyp ≥ 5.7 m/s	25	100	89	NR/NR	≥18% relative gradient (papav.)	Group C. Non-standard PSVR parameter; Kappa 0.80; hypothesis-generating
de Smet et al. (1996) [16]	PSVR ≥ 2.8	112	86	84	NR/NR	Arteriographic ≥50% + FAP pressure categories	Group D (arteriographic). Primary reference: angiography, not pressure

Group A Studies (≥10 mmHg Absolute; Directly Comparable)

For PSVR ≥2.5, Heinen et al. [13] found 83% sensitivity and 67% specificity; Coffi 2001 [15] reported 37% sensitivity and 90% specificity. This is the most methodologically valid cross-study comparison that can be found in this review because both studies have the same definition of the reference standard. This sensitivity difference is explained by spectrum restriction: Coffi's cohort enrichment for borderline lesions (PSVR 1.5-3.5) contributes to reduced sensitivity and variability in sensitivity estimates at any given threshold, consistent with spectrum bias [9]. Such restriction to borderline lesions is known to reduce sensitivity at fixed diagnostic thresholds and contributes to instability in accuracy estimates.

Group B Study (Composite Threshold)

DPSVe ≥1.4 m/s following bicycle ergometer exercise achieved 93% sensitivity and 87% specificity in 43 borderline iliac stenoses [14]. This was significantly better than resting PSVR ≥2.0 (sensitivity 75%, specificity 80%) and PSVR ≥2.5 (sensitivity 39%, specificity 93%) in the same group. The composite reference threshold (≥20 mmHg at rest or ≥15% FBI post-papaverine) is more stringent than the ≥10 mmHg Group A threshold, which means that Group B results should not be directly compared with Group A accuracy estimates. These findings should be interpreted as hypothesis-generating, as they are derived from a single study using a composite reference standard.

Group C Study (Relative Gradient)

Resting PSVR ≥2.0 showed 100% sensitivity but only 21% specificity against the ≥18% papaverine pressure gradient threshold in 25 stenoses from 23 patients [17]. However, the study included 23 patients with intentional enrichment for moderate lesions near the PSVR 2.0 boundary, and 25 segments are insufficient to generate stable sensitivity estimates (binomial 95% CI for 100% sensitivity of 85%-100%). Even the authors themselves suggested that such a tendency may be indicative of the over-sensitivity of duplex ultrasonography or under-sensitivity of papaverine testing to detect 50% diameter-reducing lesions, a methodologically important observation that cannot be confirmed in isolation and is not directly comparable to Groups A or B due to the relative reference threshold. The hyperemic velocity difference parameter (DPSVhyp) ≥5.7 m/s reported in the same study achieved 100% sensitivity and 89% specificity (Kappa 0.80) and is related to DPSVe but uses a different stimulus (papaverine) and measurement method (direct stenosis velocity versus common femoral derivation). 

Group D Study (Arteriographic Reference)

PSVR ≥2.8 yielded 86% sensitivity and 84% specificity in 112 segments from 95 unselected symptomatic patients [16]. These values are determined in comparison with arteriographic stenosis ≥50% as the primary reference, not against an isolated pressure gradient, and thus measure anatomic rather than physiological target condition. De Smet's data are preserved for contextual completeness and for comparison with the rest of the angiographic literature, not as evidence for a pressure-validated PSVR threshold.

Heinen et al. [13] reported that combining PSVR ≥2.5 with a monophasic or biphasic common femoral artery waveform achieved 94% sensitivity and 75% specificity in a post-hoc subgroup of 20 stenoses. Sensier et al. [17] reported that hyperemic common femoral pulsatility index ≤1.3 and resistance index ≤0.6 both yielded 100% sensitivity and 76% specificity (Kappa 0.60) against the papaverine pressure threshold. Both observations are hypothesis-generating only; they are based on post-hoc subgroup analyses in small, spectrum-restricted datasets, and the thresholds were applied to the same data from which they were derived without external validation.

Formal meta-analysis and forest plots could not be done without reconstructable 2 x 2 contingency tables. A table containing a structured summary of all the available sensitivity and specificity estimates by target condition group is shown in Table 5. This tabulation serves as the surrogate for visual synthesis required by PRISMA-DTA, making explicit which comparisons are methodologically legitimate (within Group A only) and which are not (across groups). No pooled estimates are provided, as combining results across target condition groups would produce a clinically meaningless composite.

**Table 5:** Structured sensitivity/specificity tabulation. Sens, sensitivity; Spec, specificity; %, percentage; nitro, nitroglycerin; papav., papaverine; FBI, femoral-brachial index; DPSVe, absolute increase in PSV after exercise.

Study [Reference]	PSVR cutoff	Segments (n)	Sens (%)	Spec (%)	Reference group	Comparability
Heinen et al. (2018) [13]	PSVR ≥2.5	35	83	67	A: ≥10 mmHg (nitro)	Limited: different stimulus (nitro); spectrum restricted
Coffi et al. (2001) [15]	PSVR ≥2.5	58	37	90	A: ≥10 mmHg (papav.)	Directly comparable to Heinen within Group A only
Coffi et al. (2002) [14]	PSVR ≥2.5	43	39	93	B: composite ≥20 mmHg/≥15% FBI	Not comparable to Group A (different threshold)
de Smet et al. (1996) [16]	PSVR ≥2.8	112	86	84	D: arteriographic ≥50%	Not comparable (anatomic reference standard)
Sensier et al. (2000) [17]	PSVR ≥2.0	25	100	21	C: ≥18% relative (papav.)	Not comparable (relative %; small n; deliberate moderate enrichment)
Coffi et al. (2002) [14]	DPSVe ≥1.4 m/s	43	93	87	B: composite ≥20 mmHg/≥15% FBI	Best-performing parameter; not PSVR-based; Group B only

Several factors precluded formal meta-analysis beyond those already described: differences in hyperemic stimuli (nitroglycerin, papaverine 20 mg, papaverine 50 mg, and exercise-induced), producing different magnitudes and time courses of vasodilation that are not physiologically interchangeable; differences in reference standard thresholds spanning four distinct definitional groups; partial patient overlap between Coffi et al. [14] and Coffi et al. [15]; differences in threshold pre-specification; and the small number of studies. Any pooled estimate from these studies would possibly lead to false clinical significance.

Using the GRADE framework, both sensitivity and specificity were found to have a very low quality of evidence indicating a risk of bias (spectrum restriction in four of five studies; post-hoc or unclear threshold specification in at least two); inconsistency (sensitivity ranges of 37%-100% for PSVR ≥2.0 and 37%-83% for PSVR ≥2.5, even when restricted to within-group comparisons); indirectness (four distinct target condition groups, meaning different studies evaluate physiologically distinct constructs; partial cohort overlap); imprecision (23-95 patients per study, no CI reported); and likely publication bias in a three-to-five publication literature [13-17]. Certainty ratings across the four target condition groups are not directly comparable due to differences in underlying reference standards and target condition definitions.

The very low GRADE rating in this case points to a more fundamental problem. GRADE for diagnostic test accuracy requires a single, clearly defined target condition and reference standard. This review applies GRADE across four conceptually heterogeneous target condition groups. That was a deliberate choice since the goal of the review is to map gaps in the evidence; documenting the full scope of definitional heterogeneity is itself a primary objective. In addition, this very low rating partly reflects the structural inadequacy of the evidence base rather than performance variability for a single well-defined clinical question. Readers should note that the most internally valid GRADE assessment applies to Group A studies only (Heinen et al. [13] and Coffi et al. [15], both using ≥10 mmHg absolute hyperemic gradient), for which certainty remains very low due to spectrum bias, small sample sizes, and absence of CI.

Discussion

This systematic review identified five small, heterogeneous studies validating PSVR cutoffs for iliac artery stenosis against a hyperemic IA pressure reference standard, four from Dutch vascular centers and one from a UK university hospital (Leicester). The most notable observation is that there is a high degree of variability in diagnostic performance at all the tested thresholds. For PSVR ≥2.5 in the two directly comparable Group A studies (both using ≥10 mmHg absolute gradient), sensitivity ranged from 37% to 83% and specificity from 67% to 90%. This variability is not random but shows inherent differences in patient selection, consistent with spectrum bias [9].

The inclusion of Sensier et al. [17] and Coffi et al. [14] extends the evidence base in two meaningful directions: Sensier et al. is the first non-Dutch study in this review, modestly broadening geographic scope, and it offers a clear mechanistic observation of the discordance between PSVR and papaverine pressure testing in the moderate disease range. The most informative single study in this review is Coffi et al. on the post-hyperemic velocity parameters, suggesting that DPSVe may outperform resting PSVR for borderline lesions in the same patient cohort [14]. All five studies consistently showed that there is no single resting PSVR threshold with sufficient sensitivity and specificity across the full range of iliac stenosis severity.

The main problem for cross-study comparability is not threshold heterogeneity but target condition heterogeneity. A gradient of 10 mmHg or above produced by nitroglycerin (nitric oxide-mediated smooth muscle relaxation) is physiologically different from the same gradient produced by papaverine (phosphodiesterase inhibition), and hyperemia produced by exercise [14] is a third physiologically different stimulus [17]. Moreover, absolute and relative thresholds measure different constructs: a 10 mmHg gradient in a hypotensive patient is not hemodynamically identical to 10 mmHg in a hypertensive patient, and a relative 18% gradient confounds the systemic pressure systematically [7]. These differences justify the four-group classification applied in this review and support the conclusion that the evidence base does not favor the use of a single pooled estimate [6-7].

The use of per-segment analysis exaggerates the apparent accuracy in all five studies. The Sensier et al. data further demonstrates the anatomy-physiology dissociation: among 15 stenoses with PSVR ≥2.0, none yielded a significant post-papaverine pressure drop, indicating that anatomic and physiologic measures of stenosis significance are more discordant in the moderate-disease range than is commonly appreciated [17]. Tetteroo et al. also reported poor correlation between duplex velocity measurements and pressure gradients following iliac intervention [18].

The results of this review are inconsistent with the broader duplex literature, which has often reported more positive accuracy for PSVR. A 1996 meta-analysis by Koelemay et al. [19], using angiographic reference standards, reported a pooled sensitivity of 86% and specificity of 97% for detecting aortoiliac disease. The substantially lower and more variable accuracy observed in the present review reflects the difference between anatomic and physiologic reference standards. A 2025 systematic review by Dias et al. showed a high specificity but variable sensitivity in the aortoiliac segment compared to arteriography, which is generally consistent with our findings [20].

The available data cannot be used to make an evidence-based recommendation on a specific PSVR cutoff due to the low quality of evidence. The most commonly studied resting threshold in the literature of a physiologic reference standard is PSVR 2.5, which is often cited in the broader duplex literature [6-7]; however, this reflects frequency of use in a small and methodologically limited literature, not demonstrated clinical validity. The 2024 ESC guidelines recommend duplex ultrasound as first-line imaging but do not specify a validated PSVR threshold for iliac stenosis, which is the same gap that is found here [1].

PSVR should not be applied alone. Combined parameters, PSVR with common femoral artery waveform analysis, and particularly post-exercise DPSVe, are physiologically reasonable and show preliminary promise, but the evidence supporting each is hypothesis-generating only. When PSVR values fall in the borderline range of approximately 1.5 to 3.5, clinical judgment and further investigations, such as exercise ankle-brachial index, computed tomography angiography, or magnetic resonance angiography, should guide decision-making rather than PSVR alone [6].

The results of this review outline essential methodological priorities for future research. Research should switch to per-patient analysis, generalized estimating equations, or cluster-robust standard errors. Thresholds must be pre-specified and prospectively registered. The vascular community should work toward consensus on a standardized hyperemic stimulus and single pressure threshold; IA papaverine or adenosine with a peak-to-peak gradient of ≥10 mmHg are good candidates. Future studies should include complete 2×2 contingency tables with CI as required by the Standards for Reporting Diagnostic Accuracy (STARD) guidelines [21]. Multicenter, large, and adequately powered studies with unselected consecutive patients at the entire range of disease severity are urgently required.

The methodological suggestions that are specific to the newly incorporated studies are: standardization of relative or absolute pressure gradient of significance; prospective evaluation of DPSVe and resting PSVR in the same unselected cohort using a single pre-defined reference standard; and avoidance of deliberate cohort enrichment for borderline lesions, which leads to spectrum bias that artificially distorts estimates of accuracy.

Clinical implications

In current clinical practice, PSVR should not be used as a standalone determinant of hemodynamically significant iliac artery stenosis. While a threshold around 2.5 is widely used, it should be interpreted as an empirical convention rather than a validated diagnostic criterion. In borderline cases (PSVR approximately 1.5-3.5), additional functional assessment, such as exercise testing, pressure measurements, or cross-sectional imaging, should guide decision-making. Duplex findings should be integrated with clinical presentation and complementary investigations rather than used in isolation.

Limitations

This review has several limitations. First, the evidence base is extremely limited (five studies, 254 patients). Second, relevant unpublished studies may have been missed; the scarcity is probably due to the actual rarity of the research based on hyperemic pressure measurement as a reference standard. Studies using angiographic or other indirect reference standards were intentionally excluded, which may limit broader contextual interpretation, but were necessary to address the specific physiologic research question. Third, the inability to reconstruct 2×2 contingency tables precluded meta-analysis and formal forest plots; the tabulated form (Table 4) is the largest possible quantitative synthesis. Fourth, the evidence base covers four different target condition groups whose reference standards are not interchangeable, restricting all cross-study comparisons to narrative synthesis. Fifth, there is partial overlap of patients between Coffi et al. [14] and Coffi et al. [15], implying that two of the five studies are not entirely independent, which may overestimate the size of the evidence base. Sixth, the five studies did not provide any CI, and all of them performed per-segment analysis, which made the estimation of the precision unreliable. Seventh, publication bias is a significant concern that should be evaluated qualitatively. The literature on this particular clinical question is sparse because only five eligible studies were found in almost thirty years (1996-2018).

## Conclusions

This systematic review and evidence-gap mapping exercise demonstrates limited and low-certainty evidence for physiologically validated PSVR thresholds for hemodynamically significant iliac artery stenosis. Both sensitivity and specificity have a very low overall certainty of evidence. PSVR ≥2.5 should be recognized as an expert-informed practice convention, not a validated diagnostic criterion. Post-hyperemic and post-exercise velocity measures appear to outperform resting PSVR for borderline lesions.

Multicenter validation studies using prospectively registered thresholds, a single standardized hyperemic stimulus, and a pressure-based reference standard, unselected consecutive patients, patient-level statistical analysis, full reporting of CI, and full adherence to the standards for reporting of diagnostic accuracy studies are urgently needed.
